# Mouse liver injury induces hepatic macrophage FGF23 production

**DOI:** 10.1371/journal.pone.0264743

**Published:** 2022-03-01

**Authors:** Pradeep Kumar, Yunshan Liu, Yang Shen, Jacquelyn J. Maher, Francesca Cingolani, Mark J. Czaja

**Affiliations:** 1 Division of Digestive Diseases, Department of Medicine, Emory University School of Medicine, Atlanta, Georgia, United States of America; 2 Department of Medicine and Liver Center, University of California, San Francisco, San Francisco, California, United States of America; University of Navarra School of Medicine and Center for Applied Medical Research (CIMA), SPAIN

## Abstract

Fibroblast growth factor 23 (FGF23) is a bone marrow cell produced hormone that functions in the intestine and kidney to regulate phosphate homeostasis. Increased serum FGF23 is a well-established predictor of mortality in renal disease, but recent findings linking increased levels to hepatic and cardiac diseases have suggested that other organs are sources of FGF23 or targets of its effects. The potential ability of the liver to produce FGF23 in response to hepatocellular injury was therefore examined. Very low levels of *Fgf23* mRNA and FGF23 protein were detected in normal mouse liver, but the amounts increased markedly during acute liver injury from the hepatotoxin carbon tetrachloride. Serum levels of intact FGF23 were elevated during liver injury from carbon tetrachloride. Chronic liver injury induced by a high fat diet or elevated bile acids also increased hepatic FGF23 levels. Stimulation of toll-like receptor (TLR) 4-driven inflammation by gut-derived lipopolysaccharide (LPS) underlies many forms of liver injury, and LPS induced *Fgf23* in the liver as well as in other organs. The LPS-inducible cytokines IL-1β and TNF increased hepatic *Fgf23* expression as did a TLR2 agonist Pam2CSK3. Analysis of *Fgf23* expression and FGF23 secretion in different hepatic cell types involved in liver injury identified the resident liver macrophage or Kupffer cell as a source of hepatic FGF23. LPS and cytokines selectively induced the hormone in these cells but not in hepatocytes or hepatic stellate cells. FGF23 failed to exert any autocrine effect on the inflammatory state of Kupffer cells but did trigger proinflammatory activation of hepatocytes. During liver injury inflammatory factors induce Kupffer cell production of FGF23 that may have a paracrine proinflammatory effect on hepatocytes. Liver-produced FGF23 may have systemic hormonal effects as well that influence diseases in in other organs.

## Introduction

Fibroblast growth factor 23 (FGF23) is a bone marrow cell produced hormone that regulates phosphate homeostasis through modulation of intestinal absorption and renal excretion. FGF23 circulates as both a full-length intact bioactive protein (iFGF23) and inactive cleaved fragments (cFGF23) [1,2]. Serum FGF23 levels are increased in chronic kidney disease in direct proportion to the loss of renal function. In addition to causing hyperphosphatemia, elevated FGF23 is associated with chronic kidney disease progression and mortality [3,4]. Risk from increased FGF23 is out of proportion to the resultant alterations in bone-mineral metabolism, suggesting that high levels of circulating FGF23 have pathophysiological effects through mechanisms other than the dysregulation of phosphate homeostasis [5]. Although the primary focus on FGF23’s effects have been in the kidney, elevated FGF23 has also been linked to disease outcomes in other organs such as the liver, heart and lung [6–10]. Defining mechanisms of excessive FGF23 production and its pathophysiological effects are of importance to a variety of tissues and the relationship of renal disease to diseases in other organs.

In addition to the standard mineral-related inducers of FGF23 which include phosphate, calcium, parathyroid hormone, and vitamin D, recent studies have implicated tissue inflammation as a cause of elevated FGF23 [11]. This inflammatory effect has been attributed to increased cleavage of iFGF23 to cFGF23 rather than from upregulation of iFGF23 production [11]. FGF23 has also been linked to inflammation by findings that the hormone may promote inflammation through stimulatory effects on immune cells [12,13].

Inflammation is critical to the outcome of many diseases including those in the liver. Most forms of liver injury are due in large part to an excessive inflammatory response [14]. Self-limited inflammation after liver injury performs the beneficial function of tissue repair, but sustained inflammation leads to hepatocyte injury and death and further stimulation of the immune response. Critical to the development of hepatic inflammation is the initiation of an innate immune response by pattern recognition receptor stimulation from gut-derived lipopolysaccharide (LPS) [15]. It is the effects of LPS that promote liver injury from hepatotoxins such as carbon tetrachloride rather than the toxin itself or its metabolites [16]. LPS-activated hepatic macrophages release proinflammatory cytokines such as tumor necrosis factor (TNF), interleukin-1β (IL-1β), and interferon-γ (IFNγ) that induce hepatocyte injury and death and further amplify inflammation [17,18]. Serum FGF23 has been shown to be increased *in vivo* by bacteria or bacterial products such as LPS [13,19–21]. The organ sources of FGF23 are unclear but have been attributed to spleen and bone [13,19–21] and not the liver [20].

Liver injury may lead to disease in other organs. The most prevalent chronic liver disease, nonalcoholic fatty liver disease (NAFLD), has been linked to diseases in many other organs [22]. NAFLD has been implicated in promoting both cardiovascular disease and chronic renal failure. The mechanisms by which hepatocellular injury could trigger disease in the heart and kidney are unknown but likely involve the release of serum factors such as the products of inflammation.

To determine whether the liver could be a source of FGF23 during inflammation, the study examined whether hepatic expression of FGF23 is induced by liver injury. In response to acute hepatotoxic liver injury and chronic diet-induced fatty liver or cholestatic injury, FGF23 hepatic gene expression and protein content and serum iFGF23 increase markedly. Analysis of specific hepatic cell types identified Kupffer cells, the resident liver macrophage, as a source of hepatic FGF23. These findings establish that liver injury increases hepatic and systemic levels of FGF23 as the result of the stimulation of Kupffer cells by inflammatory factors.

## Materials and methods

### Animals

C57BL/6J mice (#000664; Jackson Laboratory, Bar Harbor, ME) were maintained in static cages with ¼ inch corncob bedding and cotton nestlets, automatic water feeding and unlimited access to a normal chow diet (5% fat; PicoLab^®^ Rodent Diet 20 #5053; LabDiet, St. Louis, MO) under 12 h light/dark cycles. Male and female mice 10–16 weeks of age were treated with 1 ml/kg of carbon tetrachloride (CCl_4_) diluted 1:10 in mineral oil (MilliporeSigma, St. Louis, MO), as previously performed [23]. Results were identical in both sexes and combined in all analyses. After weaning male mice 8 weeks of age were fed normal diet or high-fat diet (HFD) (60% fat; Research Diets D12492; New Brunswick, NJ) for 16 weeks [24]. Male and female extracellular signal-regulated kinase 1/2 (*Erk1/2*) knockout mice were generated and oil- or tamoxifen-treated, as previously described [25]. Male C57BL/6J mice aged 10–14 weeks were administered the following dissolved in phosphate-buffered saline: intraperitoneal injections of 7.5 mg/kg of LPS (E. coli 0111:B4; MilliporeSigma) [26]; intravenous injections of 0.25 ug of TNF, 2.5 ug of IL-1β and/or 0.25 ug of IFNγ (PeproTech, Rocky Hill, NJ) [17,18]; and intravenous injections of 25 ug of Pam2CSK4 (InvivoGen, San Diego, CA). Mice were placed under deep anesthesia by isoflurane anesthesia prior to blood drawing and sacrifice by cervical dislocation. All animal studies were approved by the Animal Care and Use Committee of the Emory University School of Medicine (Protocol #PROTO201800139 and #PROTO201800146) and followed National Institutes of Health guidelines for animal care.

### Quantitative real-time reverse transcription polymerase chain reaction (qRT-PCR)

RNA extraction from whole liver or cultured cells, reverse transcription, and qRT-PCR using the primers (Integrated DNA Technologies, Coralville, IA) in Table 1 were performed as previously described [27]. Data were analyzed by the 2^-ΔΔCT^ method for relative quantification and normalized to glyceraldehyde 3-phosphate dehydrogenase.

**Table 1:** PCR primer sequences.

Gene	Forward primers (5ʹ-3ʹ)	Reverse primers (5ʹ-3ʹ)
*Ccl2*	CAGCCAGATGCAGTTAACGCCCCA	TGGGGTCAGCACAGACCTCTCTC
*Cox2*	TGCACTATGGTTACAAAAGCTGG	TCAGGAAGCTCCTTATTTCCCTT
*Crp*	TTCCCAAGGAGTCAGATACTTCC	TCAGAGCAGTGTAGAAATGGAGA
*Fgb*	CACCTGCCTCATCTTGAGCG	GCATTGACTCTGATGTCTCTCCA
*Fgf23*	CACTGCTAGAGCCTATCC	CACTGTAGATGGTCTGATGG
*Gapdh*	AGGTCGGTGTGAACGGATTTG	TGTAGACCATGTAGTTGAGGTCA
*Fgfr1*	ACTCTGGGCTGGTTGAAAAAT	GGTGGCATAGCGAACCTTGTA
*Fgfr2*	GCTATAAGGTACGAAACCAGCAC	GGTTGATGGACCCGTATTCATTC
*Fgfr3*	GCCTGCGTGCTAGTGTTCT	TACCATCCTTAGCCCAGACCG
*Fgfr4*	TGAAGAGTACCTTGACCTCCG	TCATGTCGTCTGCGAGTCAG
*Il1b*	GCAACTGTTCCTGAACTCAACT	ATCTTTTGGGGTCCGTCAACT
*Il6*	CACATGTTCTCTGGGAAATCGTGGA	TCTCTCTGAAGGACTCTGGCTTTGT
*Ifng*	ATGAACGCTACACACTGCATC	CCATCCTTTTGCCAGTTCCTC
*Kl*	TGTATGTGACAGCCAATGGAATCG	GAATACGCAAAGTAGCCACAAAGG
*Klb*	ACGACCCGACGAGGGCTGTT	GGAGGAGACCGTAAACTCGGGCTTA
*Nos2*	GTTCTCAGCCCAACAATACAAGA	GTGGACGGGTCGATGTCAC
*Tnf*	CCCTCACACTCAGATCATCTTCT	GCTACGACGTGGGCTACAG

### *Fgf23* knockdown in JS1 cells

A stable lentiviral knockdown of *Fgf23* was established in the cell line JS1 (kindly provided by Scott L, Friedman, Icahn School of Medicine at Mount Sinai, New York, NY) [28]. A plasmid encoding for an shRNA to *Fgf23* (Cat. #TRCN0000372259; sequence: CCGGTCCTCAGAGCCTATCCCAATGCTCGAGCATTGGGATAGGCTCTGAGGATTTTTG) was purchased from MilliporeSigma. To generate lentiviral particles, HEK293T cells were transfected with empty vector or the shRNA target plasmid, packaging plasmids (pRSV-Rev, pMDLg/pRRE) and envelope plasmid (pMD2.VSVG) using Fugene^®^-HD transfection reagent (Promega, Madison, WI). Viral particles were isolated and JS1 cells infected with concentrated lentiviral particles in the presence of polybrene. Stably infected polyclonal cells were selected in puromycin dihydrochloride (10 μg/ml) and maintained in puromycin (2 μg/ml) for experiments.

### Protein isolation and western blotting

Total liver or cell protein was isolated for western blotting, as previously described [29,30]. The antibodies employed were FGF23 (#MBS854462; MyBioSource, San Diego, CA) and tubulin (#2148; Cell Signaling, Danvers, MA).

### FGF23 ELISA

The concentration of iFGF23 in serum and cell culture supernatants was detected by ELISA (Immutopics Inc, San Clemente, CA) following the manufacturer’s instructions.

### Primary hepatic cell isolation

Primary Kupffer cells and hepatocytes were isolated from perfusions of mouse livers with Liberase (Roche, Indianapolis, IN), and cultured as previously described [24,26]. Hepatic stellate cells were isolated from mice by sequential perfusion with pronase (Roche) and collagenase (Crescent Chemicals, Islandia, NY). The liver was manually digested, diluted in DMEM-F12 containing 10 μg/mL DNase, and then mixed with an equal volume of DMEM-F12 containing pronase (0.2 mg/mL) and collagenase (0.5 mg/mL). The mixture was incubated in a shaking water bath at 250 rpm, 37°C for 10 min, strained through sterile gauze, washed with Gey’s Balanced Salt Solution (GBSS) containing 10 μg/mL DNase and pelleted. The pellet was resuspended in GBSS with 10 μg/mL DNase and layered atop a 9–10% Accudenz (Accurate Chemical Co., Tempe, AZ) gradient. After centrifugation at 1,400 g for 17 minutes, and hepatic stellate cells recovered at the GBSS-Accudenz interface. Cultured cells were treated with 10 ng/ml LPS, 25 ng/ml TNF, 25 ng/ml IL-1β, 100 ng/ml IFNγ (R&D Systems, Minneapolis, MN), 100 ng/ml Pam4CSK2, or 100 ng/ml FGF23 (R&D Systems) with 10 ug/ml heparin (MilliporeSigma).

### Statistical analysis

Numerical results are reported as means ± S.E. from three or more independent experiments. Statistical significance among control and treated groups was determined by one way analysis of variance (ANOVA) with Tukey post hoc corrections. Statistical significance was defined as *P*<0.05.

## Results

### Hepatic FGF23 expression is induced by toxic liver injury from CCl_4_

**Fig 1:**
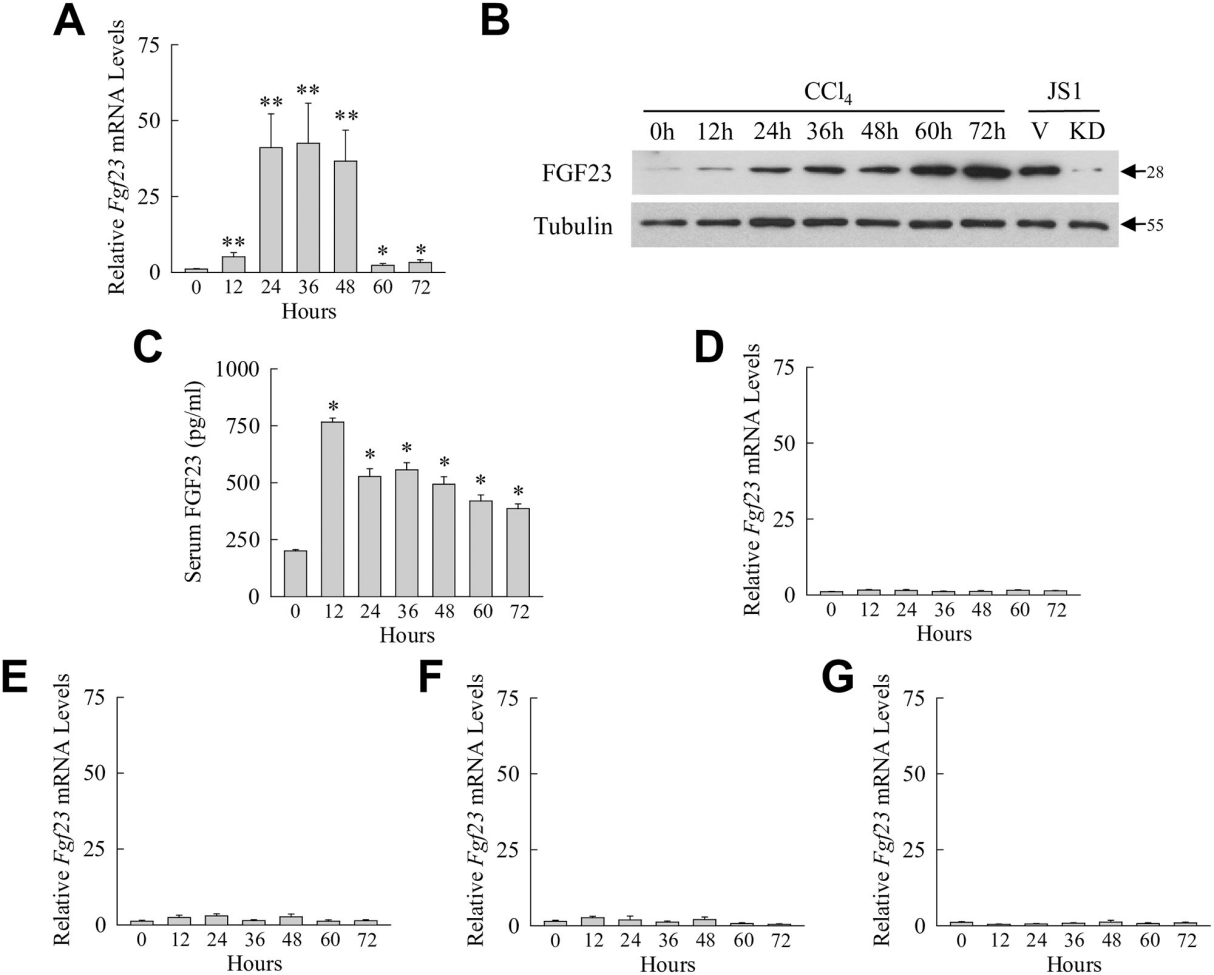
CCl_4_-induced acute liver injury results in induction of hepatic and serum FGF23. (A) Liver *Fgf23* mRNA levels in mice untreated or treated with CCl_4_ for the indicated number of hours (*P<0.05, **P<0.003 compared to untreated mice; n = 10–15). (B) Immunoblots of total liver protein from the same mice and total protein from JS1 cells infected with empty vector (V) or an shRNA to *Fgf23* (KD). Arrows indicate molecular weights in kilodaltons. Images are representative of 4 independent experiments. (C) Serum levels of iFGF23 after the indicated hours of CCl_4_ administration (*P<0.00001 compared to untreated mice; n = 6). (D-G) *Fgf23* mRNA levels in bone marrow (D), kidney (E), lung (F), and spleen (G) at the indicated times after CCl_4_ administration (n = 8–9).

To identify pathophysiological events that trigger hepatic FGF23 expression in the liver, *Fgf23* mRNA expression was assessed by qRT-PCR during acute mouse hepatic injury from the hepatotoxin CCl_4_. Levels of *Fgf23* mRNA expression were very low in normal liver but increased significantly within 12 h after CCl_4_ administration (Fig 1A). The increase in *Fgf23* gene expression was sustained throughout the period of maximal liver injury (36–48 h), and levels returned towards normal during the time of histological recovery (60–72 h) (Fig 1A). *Fgf23* gene induction by CCl_4_ resulted in increased hepatic FGF23 protein content. By immunoblotting, FGF23 was detected in low levels in control livers and increased markedly over time after CCl_4_ treatment (Fig 1B). FGF23 antibody specificity was confirmed by the demonstration of an identical size band in the FGF23-producing cell line JS1 which was decreased in cells with a lentiviral knockdown of *Fgf23* (Fig 1B).

To determine whether systemic FGF23 levels are affected by hepatic *Fgf23* induction during CCl_4_-induced liver injury, levels of iFGF23 were measured in mouse serum by ELISA. FGF23 was detectable in the serum of control mice and increased as much as 3.8-fold over 12–72 h after CCl_4_ administration (Fig 1C). Hepatotoxic liver injury resulted in hepatic FGF23 production that could have autocrine and paracrine liver effects as well as a systemic increase in circulating iFGF23 that could have additional physiological effects on extra-hepatic organs.

Increased serum FGF23 from CCl_4_ treatment may have resulted from *Fgf23* induction in organs other than liver. To examine for this possibility, *Fgf23* mRNA levels were determined in other tissues. In bone marrow, the site of the primary cell source of FGF23 production [31], *Fgf23* mRNA levels were unchanged after CCl_4_ administration (Fig 1D). Kidney, which is also a source of FGF23 [32–34], and a target of CCl_4_ toxicity [35], similarly had no significant increase in *Fgf23* mRNA (Fig 1E). Induction of *Fgf23* mRNA also failed to occur in the lung (Fig 1F), another tissue affected by the toxic effects of CCl_4_ [35], and the spleen, a reticular endothelium organ like the liver (Fig 1G). These findings indicate that injured liver is the sole source of the sustained elevation in serum iFGF23 during CCl_4_-induced hepatic injury.

### Chronic fatty and cholestatic liver injury are associated with increased hepatic FGF23

**Fig 2:**
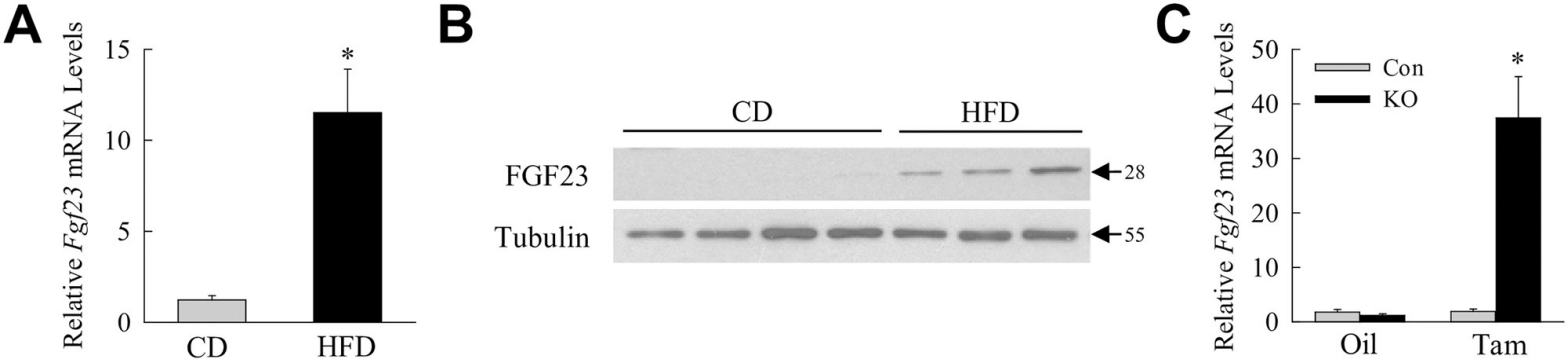
FGF23 expression is increased in chronic diet-induced fatty liver and in cholestatic liver disease. (A) *Fgf23* mRNA levels in the livers of chow diet (CD) and high fat diet (HFD) fed mice (*P<0.002 compared to chow diet; n = 7). (B) Immunoblots of total liver protein from the same mice probed for FGF23 and tubulin. Molecular weights in kilodaltons are indicated by arrows. Images are representative of 3 independent experiments. (C) *Fgf23* mRNA levels in littermate control (Con) and *Erk1/2-knockout* (KO) mice treated with oil or tamoxifen (Tam) (*P*<0.0003 compared to all other groups; n = 11–13).

To determine whether hepatic FGF23 production is a general response to liver injury, FGF23 levels were examined in two murine models of chronic liver injury distinct from that of acute CCl_4_ administration. In an HFD-induced mouse model of NAFLD, *Fgf23* mRNA expression was induced greater than 10-fold in the livers of HFD-fed mice (Fig 2A). Elevated levels of FGF23 protein were also detected in the livers of HFD-fed mice by immunoblotting (Fig 2B). *Fgf23* expression was increased 37-fold in the livers of tamoxifen-injected *Erk1/2*-knockout mice that develop chronic bile acid-induced cholestatic liver injury and fibrosis (Fig 2C) [25]. Hepatic FGF23 production is induced in the liver in response to diverse injurious stimuli and in both acute and chronic liver injury.

### LPS induces sustained hepatic *Fgf23* expression

**Fig 3:**
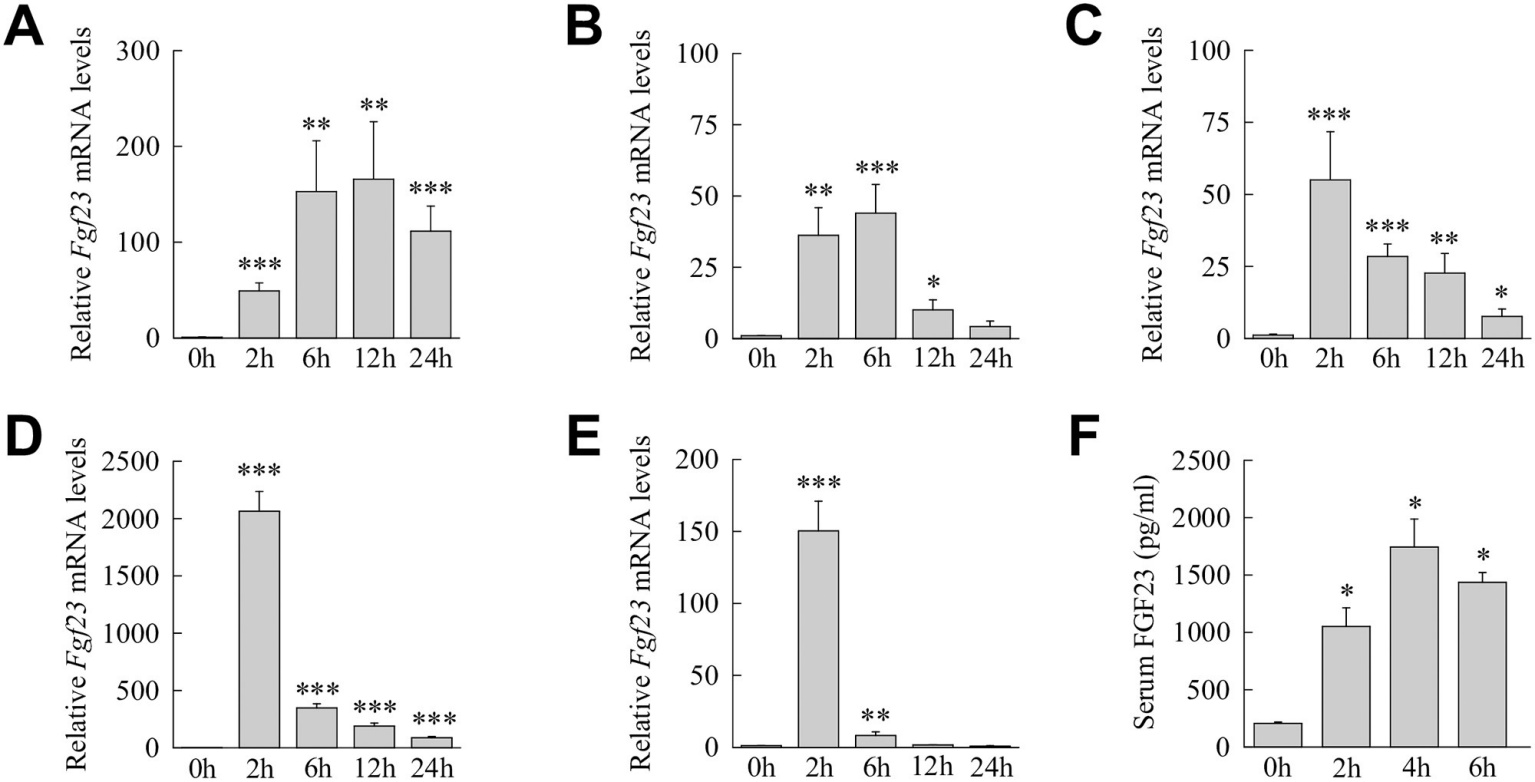
*Fgf23* is induced in the liver and other tissues in response to LPS. (A-E) *Fgf23* mRNA levels in untreated mice and mice treated for the indicated hours with LPS in liver (A), bone marrow (B), kidney (C), lung (D) and spleen (E) (*P<0.05, **P<0.01, ***P<0.001 compared to untreated mice; n = 8–11). (F) Serum iFGF23 levels in the mice (*P<0.001 compared to untreated mice; n = 5).

Most forms of liver injury, including that from CCl_4_ and HFD feeding, are dependent on hepatic effects of gut-derived bacterial products including LPS [16,36]. This fact, together with reports in nonhepatic cell types that *Fgf23* can be induced by LPS and other proinflammatory factors [11], suggested that LPS and/or its downstream effector cytokines may stimulate hepatic *Fgf23* expression. The effect of systemic LPS on *Fgf23* expression in the liver was therefore determined and compared to other organs. Liver *Fgf23* levels rose significantly by 2 h, peaked within 6 h after LPS administration and remained markedly elevated over 24 h (Fig 3A). In bone marrow there was an increase over 2–6 h with a reduction by 12 h and a return to baseline expression at 24 h (Fig 3B). Kidney demonstrated a rapid 2 h peak in expression and then *Fgf23* expression decreased although levels remained significantly elevated for 24 h (Fig 3C). Lung and spleen had similarly marked *Fgf23* induction at 2 h and levels that decreased dramatically but remained elevated at 6 h (Fig 3D and 3E). LPS led to FGF23 protein production as serum levels of iFGF23 were significantly increased after LPS administration (Fig 3F). Thus, the liver is one of multiple organs in which *Fgf23* induction occurs in response to the inflammatory stimulus of LPS and one in which expression remains sustained at high levels relative to other organs.

### Liver *Fgf23* is induced by proinflammatory factors

**Fig 4:**
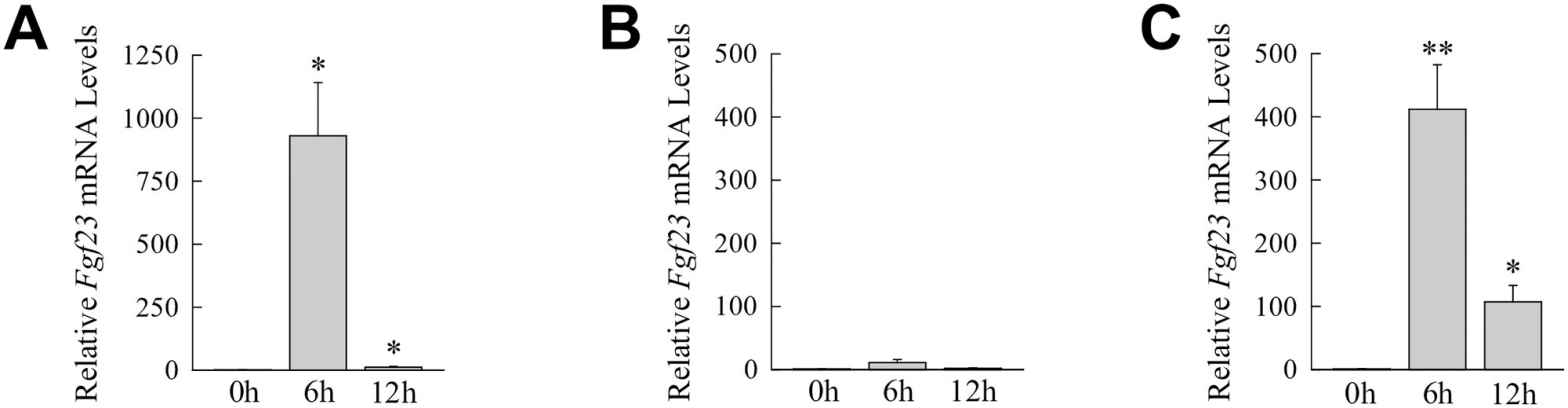
Liver *Fgf23* is induced by TNF/IL-1β. Hepatic *Fgf23* mRNA levels in untreated mice and mice 6 h and 12 h after injection with TNF/IL-1β (A), IFNγ (B) or Pam2CSK4 (C) (*P<0.01, **P<0.0001 compared to untreated mice; n = 5–9).

To identify the downstream mediators of *Fgf23* induction by LPS-initiated toll-like receptor (TLR) 4 signaling, the ability of LPS-inducible proinflammatory cytokines to increase *Fgf23* expression in the liver was examined. Mice were injected with cytokine concentrations previously determined to be biologically active in the liver [17,18]. Administration of either of the two principal LPS-inducible cytokines, TNF and IL-1β, failed individually to increase liver *Fgf23* mRNA expression (S1 Fig). However, combined treatment with TNF and IL-1β led to a marked induction of hepatic *Fgf23* gene expression (Fig 4A). In contrast, the LPS-inducible proinflammatory cytokine IFNγ failed to significantly affect *Fgf23* levels (Fig 4B).

Induction of *Fgf23* by TLR4 may be selective for this pattern recognition receptor or other TLRs may stimulate *Fgf23* expression during liver injury. In addition to the involvement of TLR4 in liver disease, TLR2 signaling contributes to the pathogenesis of liver injury [37]. Administration of the TLR2 agonist Pam2CSK4 induced liver *Fgf23* gene expression (Fig 4C). Hepatic *Fgf23* expression is therefore under the regulation of redundant proinflammatory TLR signaling pathways that can activate this gene during liver injury.

### Kupffer cells but not hepatocytes or hepatic stellate cells express FGF23

**Fig 5:**
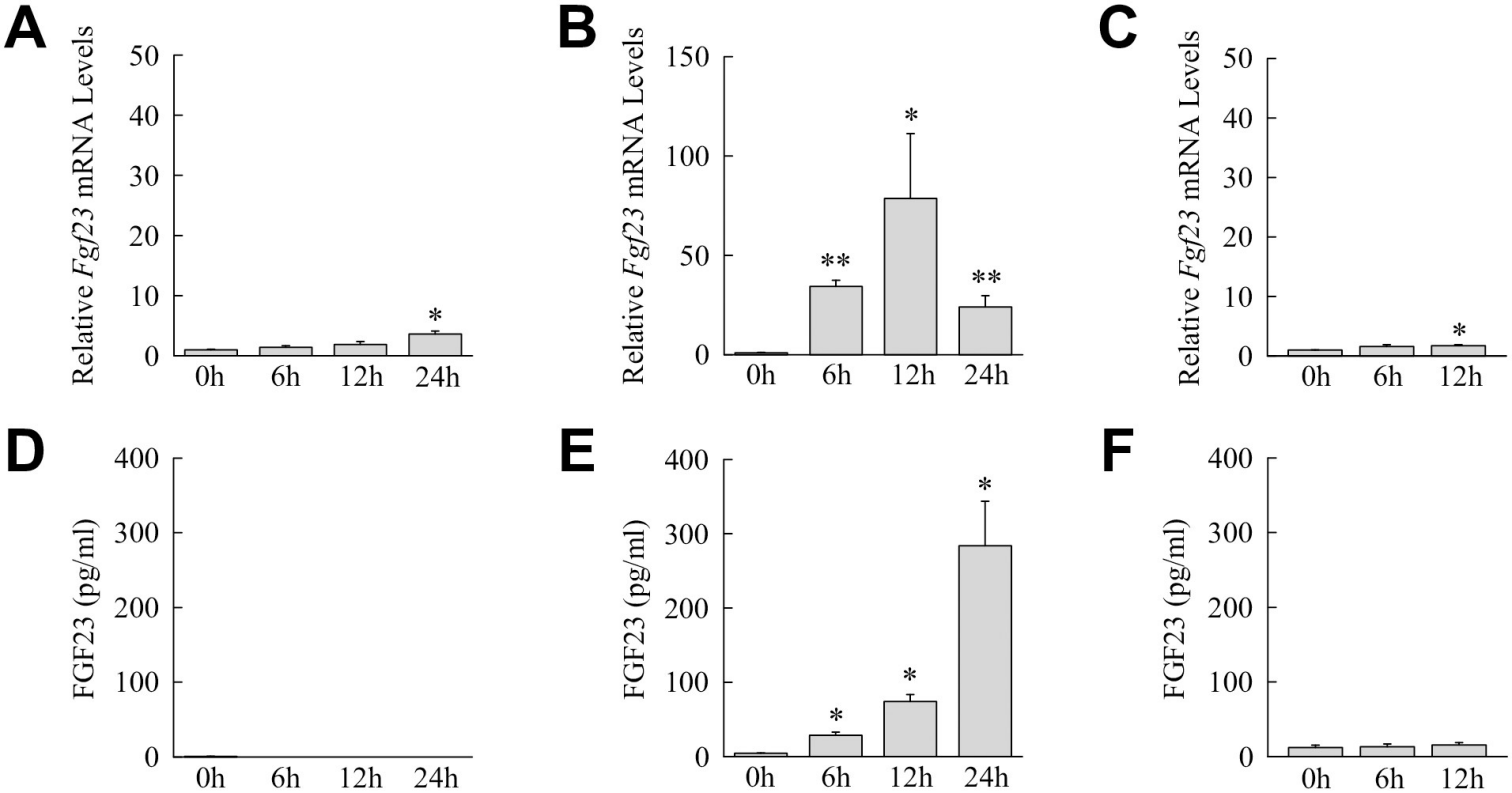
Kupffer cells are a source of hepatic FGF23. (A) Relative *Fgf23* mRNA levels in cultured mouse hepatocytes untreated or treated with LPS for the indicated hours (*P<0.0001 compared to untreated cells; n = 12). (B) *Fgf23* mRNA levels in LPS-treated Kupffer cells (*P<0.01, **P<0.0001 compared to untreated cells; n = 9). (C) *Fgf23* mRNA content in LPS-treated hepatic stellate cells (*P<0.01 compared to untreated cells; n = 5–6). (D) iFGF23 protein levels by ELISA in the culture medium of mouse hepatocytes after LPS treatment (n = 4–6). (E) iFGF23 protein in the medium of Kupffer cells (*P<0.001 compared to untreated cells; n = 8–9). (F) iFGF23 levels in hepatic stellate cell medium (n = 5).

To define the hepatic cell type(s) that produces FGF23 during liver injury, the three principal cell types involved in liver injury, hepatocytes, Kupffer cells and hepatic stellate cells, were isolated and examined *in vitro* for *Fgf23* mRNA baseline expression and induction by LPS. All three hepatic cell types had very low constitutive *Fgf23* mRNA levels. In mouse hepatocytes LPS induced only a late and modest 2.5-fold increase in *Fgf23* mRNA levels at 24 h (Fig 5A). In contrast, Kupffer cells had a sustained LPS induction of *Fgf23* over 24 h that peaked at 75-fold at 12 h (Fig 5B). Primary hepatic stellate cells had a minor 2-fold increase over their low basal levels of *Fgf23* expression at 12 h after LPS treatment (Fig 5C). Significant LPS induction of *Fgf23* gene expression is therefore confined to Kupffer cells among these three hepatic cell types.

To further validate that Kupffer cells are a source of liver FGF23, medium from the three cell types untreated and LPS-treated was assayed for levels of iFGF23 by ELISA. No iFGF23 was detected in the medium of hepatocytes untreated or LPS-stimulated (Fig 5D). Kupffer cells constitutively secreted low amounts of iFGF23 that increased 100-fold with LPS treatment (Fig 5E). Primary hepatic stellate cells produced low amounts of iFGF23 that were unaffected by LPS stimulation (Fig 5F). These low levels of iFGF23 production in hepatic stellate cells are likely secondary to the Kupffer cell contamination present in these cultures.

**Fig 6:**
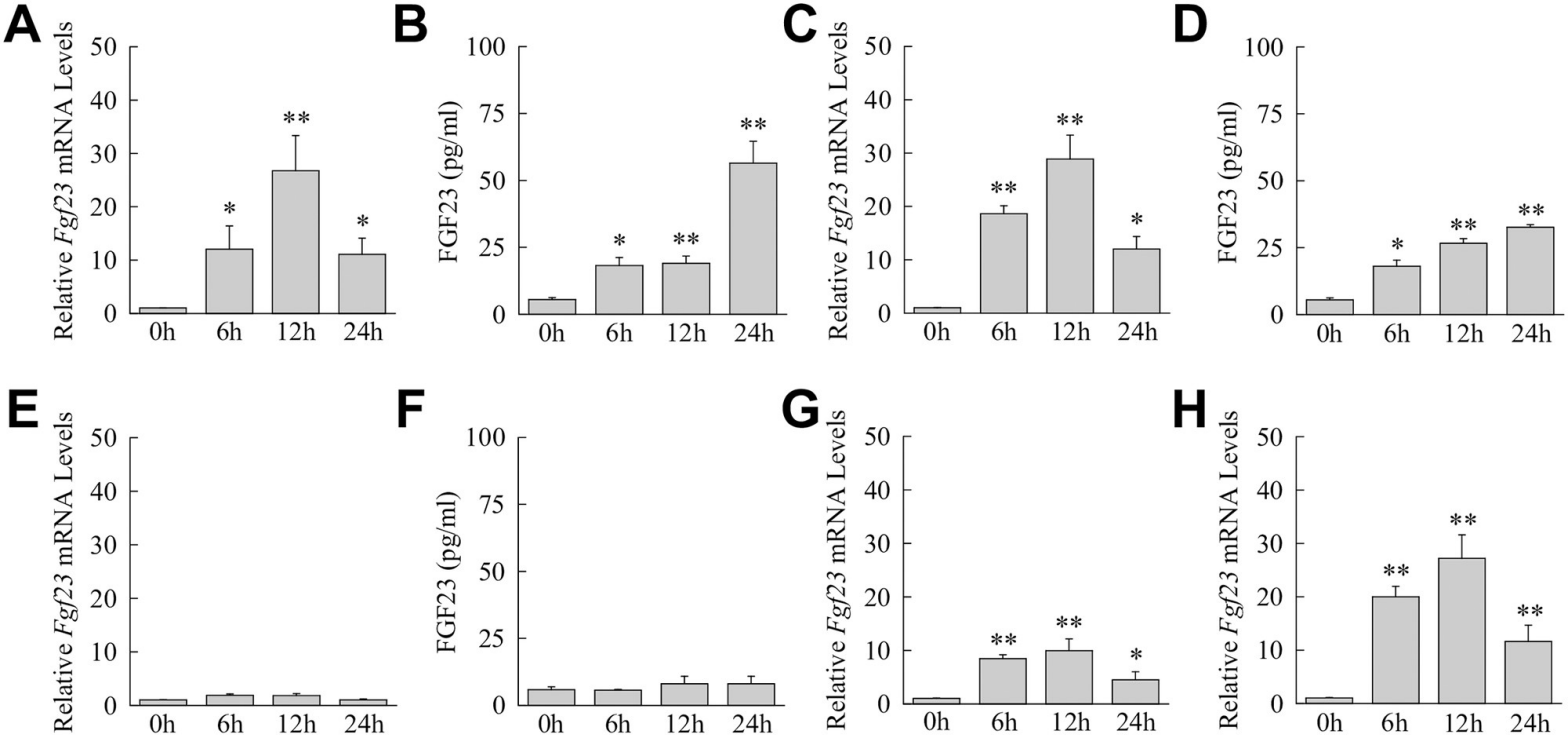
*Fgf23* is induced in Kupffer cells and bone marrow-derived macrophages in response to proinflammatory factors. (A) Relative *Fgf23* mRNA levels in Kupffer cells untreated or treated with TNF/IL-1β for the indicated hours (*P<0.01, **P<0.001 compared to untreated cells; n = 9–10). (B) iFGF23 levels in the medium of the same TNF/IL-1β-treated cells (*P<0.01, **P<0.001 compared to untreated cells; n = 6–7). (C) *Fgf23* mRNA levels in Kupffer cells treated with Pam2CSK4 (*P<0.001, **P<0.0001 compared to untreated cells; n = 8–9). (D) Medium protein levels from the same Pam2CSK4-treated cells (*P<0.01, **P<0.000001 compared to untreated cells; n = 6). (E) *Fgf23* mRNA levels with IFNγ treatment (n = 5–7). (F) iFGF23 levels in the medium with IFNγ treatment (n = 5). (G-H) *Fgf23* mRNA levels in bone marrow-derived macrophages treated with TNF/IL-1β (G) or Pam2CSK4 (H) (*P<0.02, **P<0.001 compared to untreated cells; n = 8–10).

Kupffer cells were further examined for their ability to mimic the hepatic induction of *Fgf23* by proinflammatory cytokines and TLR2 stimulation. Cultured cells had a significant induction in *Fgf23* mRNA levels (Fig 6A) and iFGF23 protein production (Fig 6B) with TNF/IL-1β treatment. Similarly, mRNA and protein induction occurred in response to the TLR2 agonist Pam2CSK4 (Fig 6C and 6D). Identical to findings in mouse liver, *Fgf23* was not induced by IFNγ in Kupffer cells (Fig 6E and 6F). Kupffer cells therefore mimic the FGF23 induction pattern of mouse liver to inflammatory factors providing further evidence that these cells are a source of FGF23 during toxic liver injury.

To determine whether TLR4/TLR2 induction of *Fgf23* was specific to Kupffer cells or a property of all macrophages, *Fgf23* expression in response to TLR stimulation was examined in bone marrow-derived macrophages. *Fgf23* mRNA levels increased in bone marrow-derived macrophages in response to TNF/IL-1β/ (Fig 6G) and Pam2CSK4 (Fig 6H). TLR4/2-dependent induction of *Fgf23* is therefore common to macrophages and not Kupffer cell specific.

### FGF23 regulates the proinflammatory phenotype of hepatocytes but not Kupffer cells

**Fig 7:**
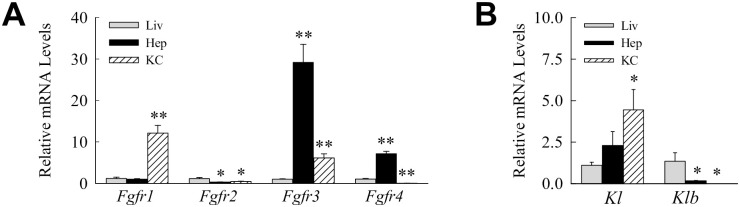
Relative levels of *Fgfr* and *Klotho* gene expression. (A) mRNA levels for the *Fgfr* genes in total liver (Liv), and isolated hepatocytes (Hep) and Kupffer cells (KC) (*P<0.03, **P<0.001 compared to liver; n = 6–7). (B) Expression levels for α- (*Kl*) and β-Klotho (*Klb*) in the same samples (*P<0.03 compared to liver; n = 6–7).

Cellular responsiveness to FGF23 is mediated by the family of FGF receptors (FGFRs) and a coreceptor Klotho [31]. Of the four FGFRs, FGFR1, FGFR3 and FGFR4 have been implicated in the transduction of FGF23 signaling in different cell types [38,39]. In some tissues signal initiation requires α- or β-Klotho as a coreceptor [40]. To define potential FGF23 target cells in the liver, the level of gene expression of these receptors/coreceptors was examined in hepatocytes and Kupffer cells relative to whole liver. Differential receptor expression exists in hepatocytes and Kupffer cells. Mouse hepatocytes have higher expression of *Fgfr3* and to a lesser extent of *Fgfr4* with low amounts of *Fgfr2* (Fig 7A). In contrast, Kupffer cells express higher levels of *Fgfr1*, lower levels of *Fgfr3*, and no *Fgfr4* (Fig 7A). *α-Klotho* mRNA is barely detectable in liver and levels are modestly higher in Kupffer cells (Fig 7B). *β-Klotho* expression is similarly very low in liver as well as in both hepatocytes and Kupffer cells (Fig 7B).

**Fig 8:**
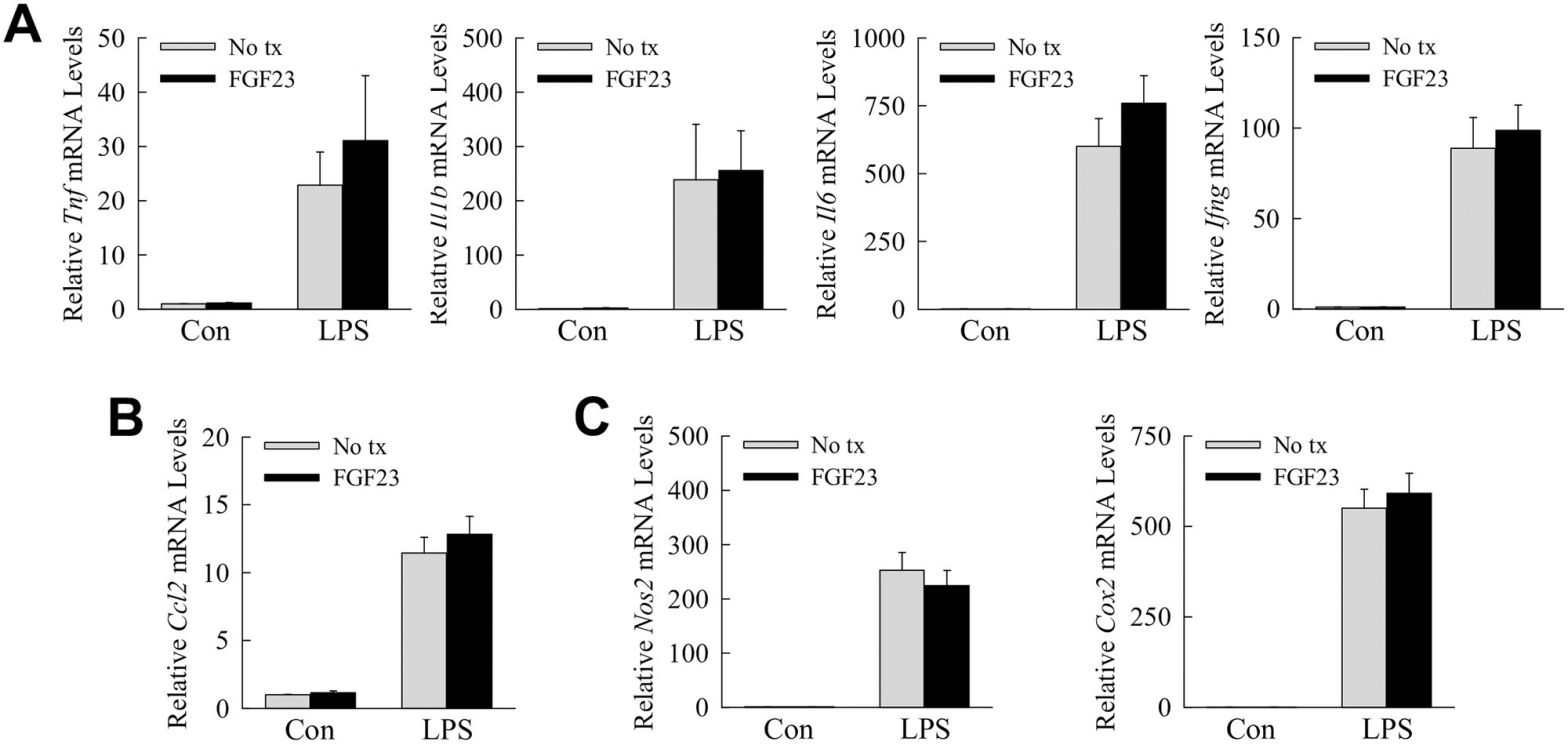
The inflammatory state of Kupffer cells untreated or LPS-treated is unaffected by FGF23. (A-C) Primary mouse Kupffer cells were untreated (No tx) or pre-treated with FGF23, and 1 h later some cells were untreated (Con) or treated with LPS. Cells were harvested 6 h after LPS treatment. Relative mRNA levels for the indicated genes are shown (n = 8–10).

The induction of hepatic FGF23 by inflammatory mediators, the ability of this factor to regulate inflammation in nonhepatic immune cells, and the presence of FGFRs in Kupffer cells suggested that FGF23 may have an autocrine effect on the inflammatory state of these cells. FGF23 treatment had no effect on basal Kupffer cell expression of the proinflammatory cytokine genes *Tnf*, *Il1b*, *Il6* and *Ifng* (Fig 8A). FGF23 also failed to alter the induction of these genes by LPS (Fig 8A). FGF23 similarly failed to affect expression of *chemokine (C-C motif) ligand 2* (*Ccl2*) (Fig 7B), or the proinflammatory genes *nitric oxide synthase* (*Nos2*) and *cyclooxygenase-2* (*Cox2*) (Fig 8C). FGF23 had no effect on basal or LPS-stimulated inflammatory Kupffer cell activation.

**Fig 9:**
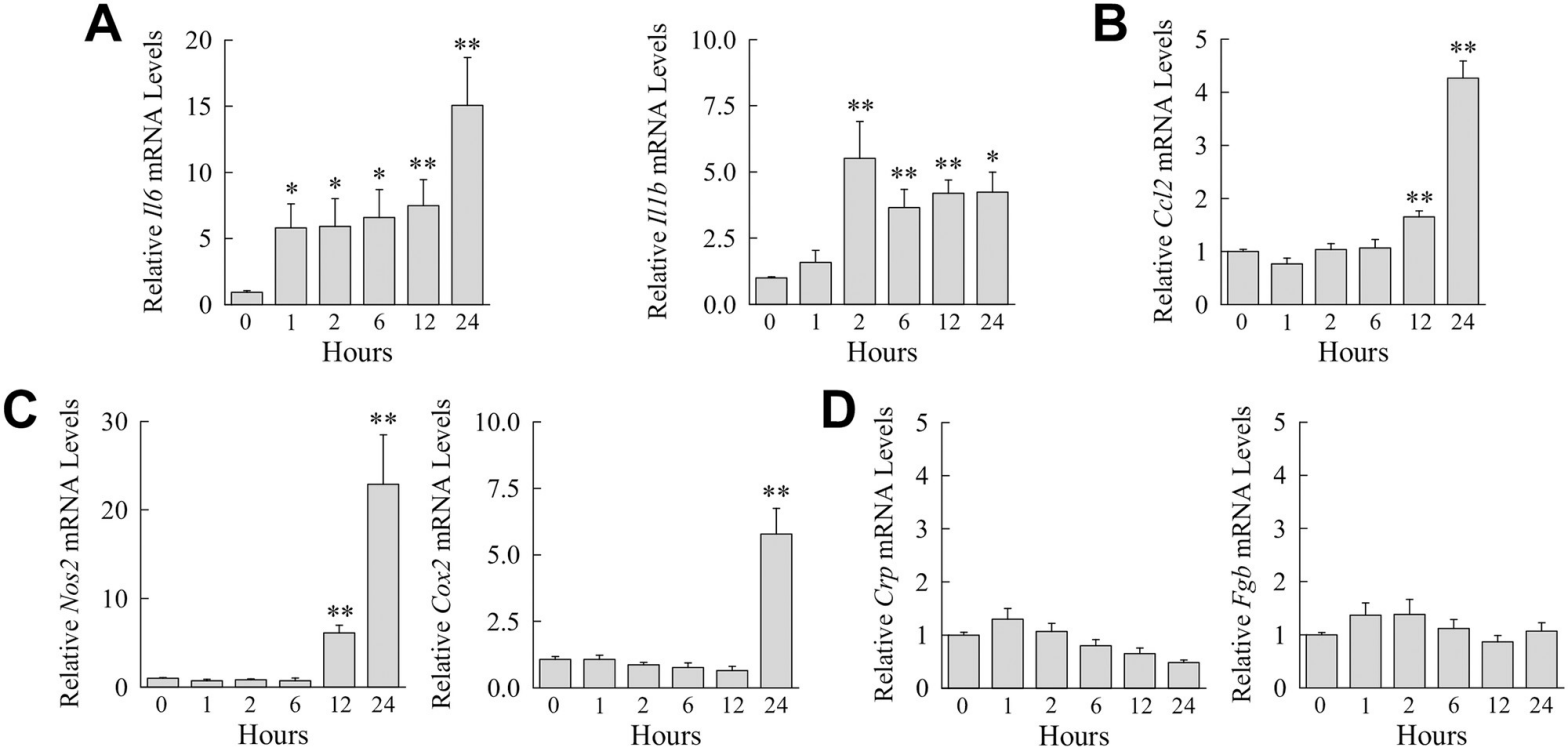
FGF23 induces a proinflammatory hepatocyte phenotype. (A-C) Primary mouse hepatocytes were untreated or administered FGF23 for the number of hours shown. Relative mRNA levels for the indicated genes including *c-reactive protein* (*Crp*) and *fibrinogen* (*Fgb*) are indicated (*P<0.05, **P<0.01 compared to untreated cells; n = 6).

Although Kupffer cells are the main cellular regulator of the hepatic immune response, hepatocytes also contribute to liver inflammation [41]. FGF23 has been reported to induce IL-6, a cytokine with mixed proinflammatory and anti-inflammatory hepatic effects, in hepatocytes [42]. Kupffer cell generated FGF23 may therefore regulate hepatic inflammation through a paracrine hepatocyte effect. FGF23 induced hepatocyte gene expression for the cytokines *Il6* and *Il1b* (Fig 9A), the chemokine *Ccl2* (Fig 9B), and the proinflammatory genes *Nos2* and *Cox2* (Fig 9C). In contrast, FGF23 failed to induce expression for the acute phase reactants *c-reactive protein* and *fibrinogen* (Fig 9D). FGF23 therefore induced a specific proinflammatory activation in primary hepatocytes.

## Discussion

FGF23 is produced primarily by bone for the function of regulating total body phosphorous homeostasis. There is a long-standing and well-established relationship of diseases of the kidney, the critical site for phosphate excretion, with elevated serum FGF23 levels. However, increases in FGF23 have more recently also been linked to diseases and their outcomes in a number of organs outside the bone-kidney axis of mineral regulation including liver, lung and heart [6–8,10,43–45]. Whether these organs are a source of FGF23 and/or are responsive to this hormone are unclear. This study demonstrates that acute and chronic liver injury induces hepatic production of FGF23 through the stimulation of the resident liver macrophages or Kupffer cells by injury-associated inflammatory factors.

Our findings demonstrate that the liver produces FGF23 in response to the diverse injuries of a hepatotoxin CCl_4_, diet-induced fatty liver, and bile acid-induced cholestatic liver disease. Hepatic FGF23 was generated rapidly by acute liver injury from CCl_4_, and its production was sustained in chronic NAFLD and cholestatic injury as demonstrated by both increased gene expression and protein content in the liver. FGF23 generation by acute liver injury from CCl_4_ was sufficient to elevate serum iFGF23 to levels comparable to that achieved by systemic LPS administration. Findings that *Fgf23* induction from CCl_4_ occurred only in the liver, and not in other tissues known to produce FGF23, indicate that the injured liver accounted for the increase in serum iFGF23 and can be a major source of excessive systemic FGF23. Increased serum FGF23 has been reported in human NAFLD [7], and in patients with chronic liver disease awaiting liver transplant [6]. Whether liver was the source of FGF23 in these conditions was not examined, but our mouse studies suggest that the liver itself could be the tissue responsible for FGF23 production in human liver diseases.

Critical to the outcome from liver injury is the extent of the associated hepatic innate immune response stimulated by intestinal bacterial products including LPS and regulated by cytokines. In contrast to several prior findings of increased serum cFGF23 but not iFGF23 during endotoxemia or bacteremia [19,21], our study demonstrates that systemic LPS administration increased serum iFGF23 levels consistent with the findings of Masuda et al. [13]. The results demonstrate that the liver is a source of FGF23 during endotoxemia whereas others have concluded that principally spleen, and to a lesser extent other tissues including bone and thymus, are the organs that produce FGF23 in response to circulating LPS [13,19,21]. Interestingly, liver was the one organ with sustained *Fgf23* induction for 24 h after LPS administration whereas mRNA levels in all other tissues dropped precipitously within 6–12 h. Liver may therefore be a major source of FGF23 during systemic inflammation. *Fgf23* induction occurred in nonhepatic tissues with LPS but not CCl_4_ injection even though CCl_4_-induced liver injury is LPS dependent [16]. This difference could reflect the fact that with CCl_4_ LPS delivery is restricted largely to the liver as the source is endogenous intestinal LPS that travels by the portal circulation directly to the liver in contrast to the systemic uptake from an intraperitoneal injection of exogenous LPS. Hepatic FGF23 expression is induced by TLR2 as well as LPS-induced TLR4 stimulation and by the proinflammatory cytokines TNF/IL1-β which may be the downstream effectors of LPS/TLR4 signaling. The fact that multiple inducers of hepatic FGF23 are proinflammatory stimuli clearly link FGF23 to the liver inflammatory response in keeping with recent evidence that this hormone may regulate inflammation.

The principal cell source of FGF23 is the bone osteoblast consistent with this protein’s known involvement with phosphorous homeostasis [31]. FGF23 expression has also been demonstrated in normal brain [46], and in renal tubule cells in a variety of forms of kidney injury [32–34]. Both the absence and presence of FGF23 expression has been reported in vascular smooth muscle cells [34,47]. Macrophages and dendritic cells have also been demonstrated to produce FGF23 [12,13]. Our findings extend the known cellular sources of FGF23 to hepatic Kupffer cells further strengthening the relationship between inflammation and this hormone.

Similar to findings with renal diseases, increases in systemic FGF23 have been linked to a poor prognosis in liver disease. Increased human FGF23 levels have been shown to predict mortality in patients waiting for liver transplant [6], and are associated with progression of hepatocellular carcinoma [48]. How FGF23 may modulate liver disease and its outcome are unclear. Autocrine/paracrine actions of FGF23 in the liver are possible as the studies demonstrate differential *Fgfr* isoform expression in hepatocytes and Kupffer cells. Given the growing association of FGF23 with inflammation, and the presence of FGFRs in these two hepatic cell types, a function for FGF23 in the regulation of the inflammatory state of these cells was examined. Although proinflammatory activation of the macrophage cell line RAW264.7 by FGF23 has been reported [12], no pro- or anti-inflammatory effect of FGF23 was detected in Kupffer cells. Hepatic FGF23 does not function in an autocrine fashion to modulate the hepatic macrophage inflammatory response. Prior studies have indicated that FGF23 induces c-reactive protein and IL-6, a cytokine with pro- and anti-inflammatory functions in the liver, in cultured hepatocytes [42]. Our findings in hepatocytes indicate that FGF23 causes a proinflammatory activation of these cells with an induction of cytokines and chemokines, but no increase in acute phase reactants such as c-reactive protein was detected. One function of FGF23 in liver injury may therefore be the promotion of the hepatocyte inflammatory response. A determination of additional effects of liver-produced FGF23 during liver injury will require further study.

Hepatic diseases are often linked to effects in other organ but the mechanisms of crosstalk between the injured liver and other tissues are largely unknown. A prime example of the effects of injury in the liver on other organs is the association of NAFLD with cardiovascular and renal disease [22]. We have demonstrated *Fgf23* induction in a mouse model of NAFLD and increased serum FGF23 levels have been found in human NAFLD [7,43]. Liver-produced FGF23 may therefore have endocrine effects in these extrahepatic diseases. Patients with NAFLD have an increased prevalence of cardiovascular disease which is the major cause of their increased mortality [49]. Increased FGF23 levels have been linked to cardiovascular disease and mortality [45]. Hepatic FGF23 generated in the setting of a fatty liver may therefore be a mechanism of cardiovascular disease in NAFLD. Our findings of hepatic FGF23 production therefore suggest a novel mechanism by which injury in the liver may adversely affect other organs. In addition, the promotion of inflammation in nonhepatic organs by hepatic FGF23 may result in the release of other proinflammatory factors from these organs which in turn trigger the progression of liver disease. The interesting possibility of the hormonal effects of hepatic FGF23 mediating the crosstalk between liver and other organs in hepatic diseases requires further study.

The investigations demonstrate that increased serum FGF23 levels do not simply reflect renal dysfunction or phosphate retention but may result from hepatic pathophysiology. Acute or chronic liver injury leads to hepatic production of FGF23 through the effects of proinflammatory factors such as LPS and cytokines on hepatic macrophages. The studies identify FGF23 as a potential new autocrine or paracrine regulator of liver disease. The findings also uncover a new possible hormonal mediator of crosstalk between an injured liver and extrahepatic organs that may be a mechanism of disease in distal tissues or explain the ability of these other organs to promote liver injury.

## Supporting information

S1 Fig TNF and IL-1β fail to induce hepatic *Fgf23* expression. Hepatic *Fgf23* mRNA levels in untreated mice and mice 6 h and 12 h after injection with TNF (A), or IL-1β (B) (n = 4–5).
(PDF)

S2 Fig Unaltered immunoblot images. Unaltered radiographic images for Figs 1B and 2B.
(PDF)
