# Supplementary figures and images for: Mouse liver injury induces hepatic macrophage FGF23 production
Source: PLoS One. 2022 Mar 1;17(3):e0264743. doi: 10.1371/journal.pone.0264743 (PMC8887750; doi:10.1371/journal.pone.0264743)

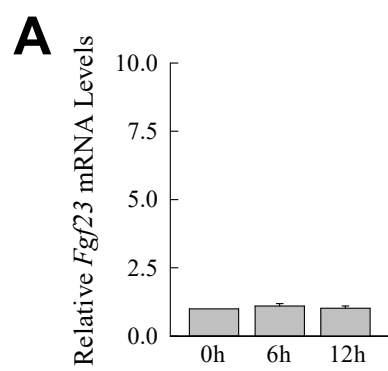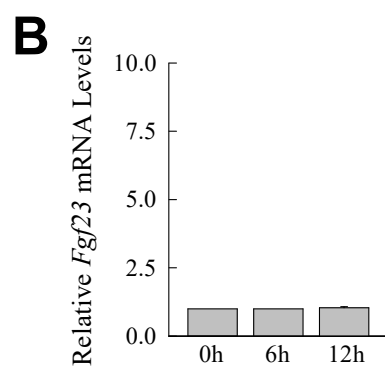

Fig S1

Supplement: S1 Fig — Hepatic Fgf23 mRNA levels in untreated mice and mice 6 h and 12 h after injection with TNF (A), or IL-1β (B) (n = 4–5). (PDF) [file pone.0264743.s001.pdf]
