# Supplementary material for: Mouse liver injury induces hepatic macrophage FGF23 production
Source: PLoS One. 2022 Mar 1;17(3):e0264743. doi: 10.1371/journal.pone.0264743 (PMC8887750; doi:10.1371/journal.pone.0264743)

Fig. 1B original full size western film images for FGF23 (top panel) and tubulin (bottom panel). Numbers represent molecular weight marker sizes in KD.

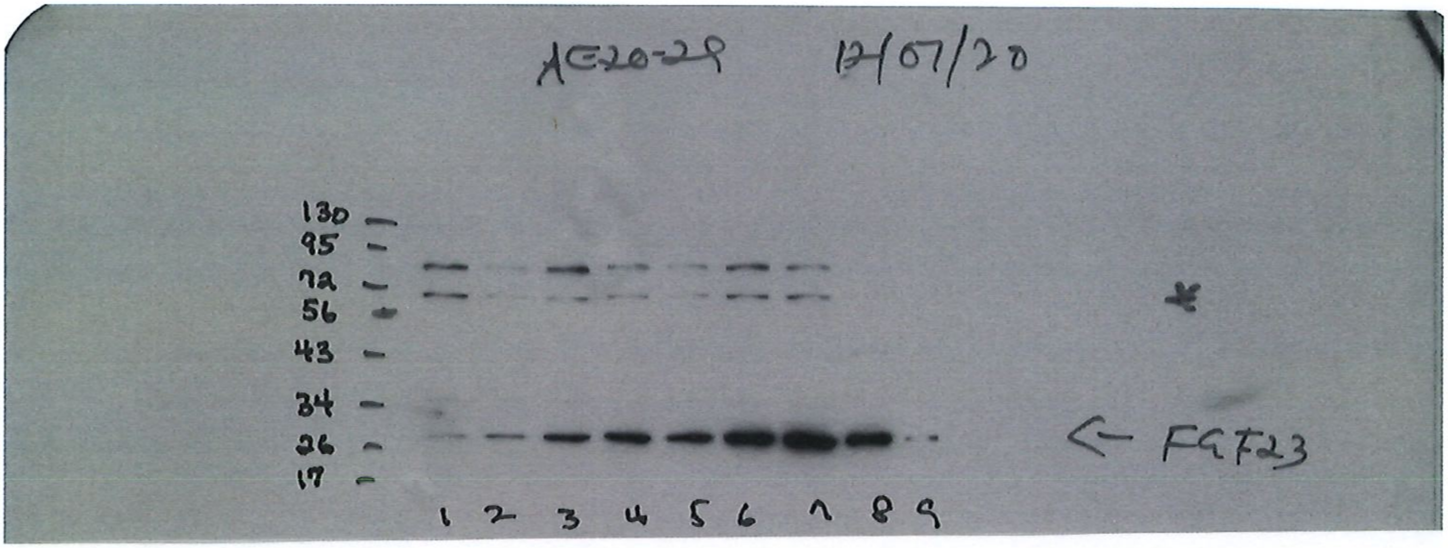

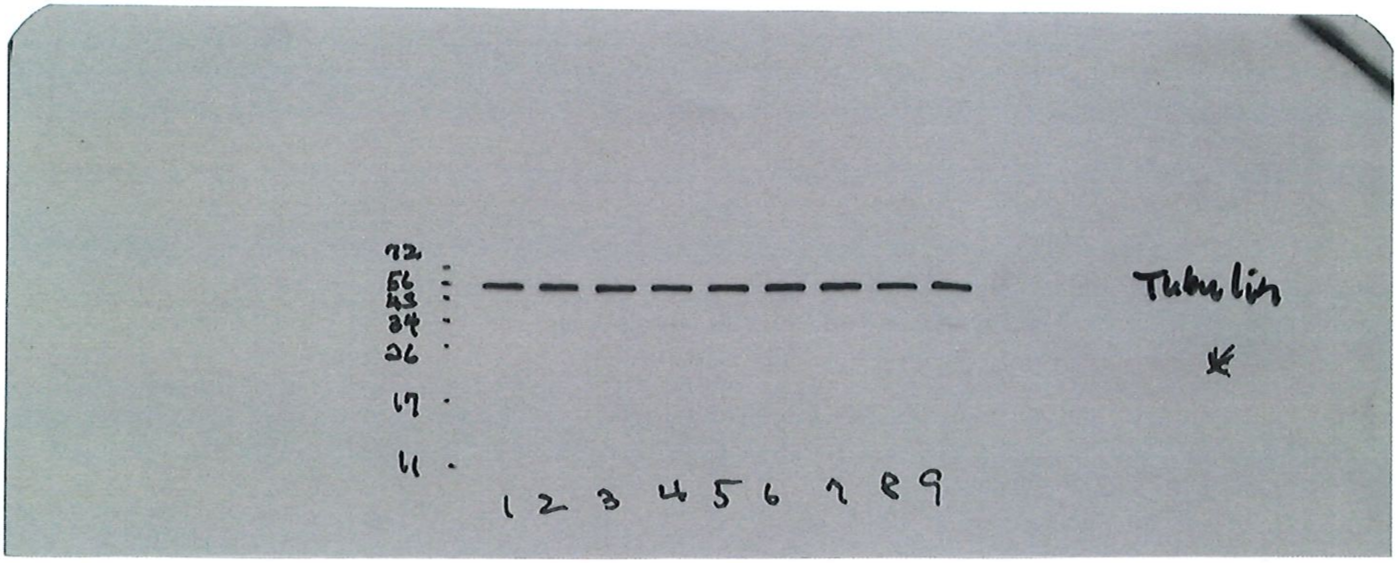

Fig. 2B original full size western film images for FGF23 (top panel) and tubulin (bottom panel). Numbers represent molecular weight marker sizes in KD.

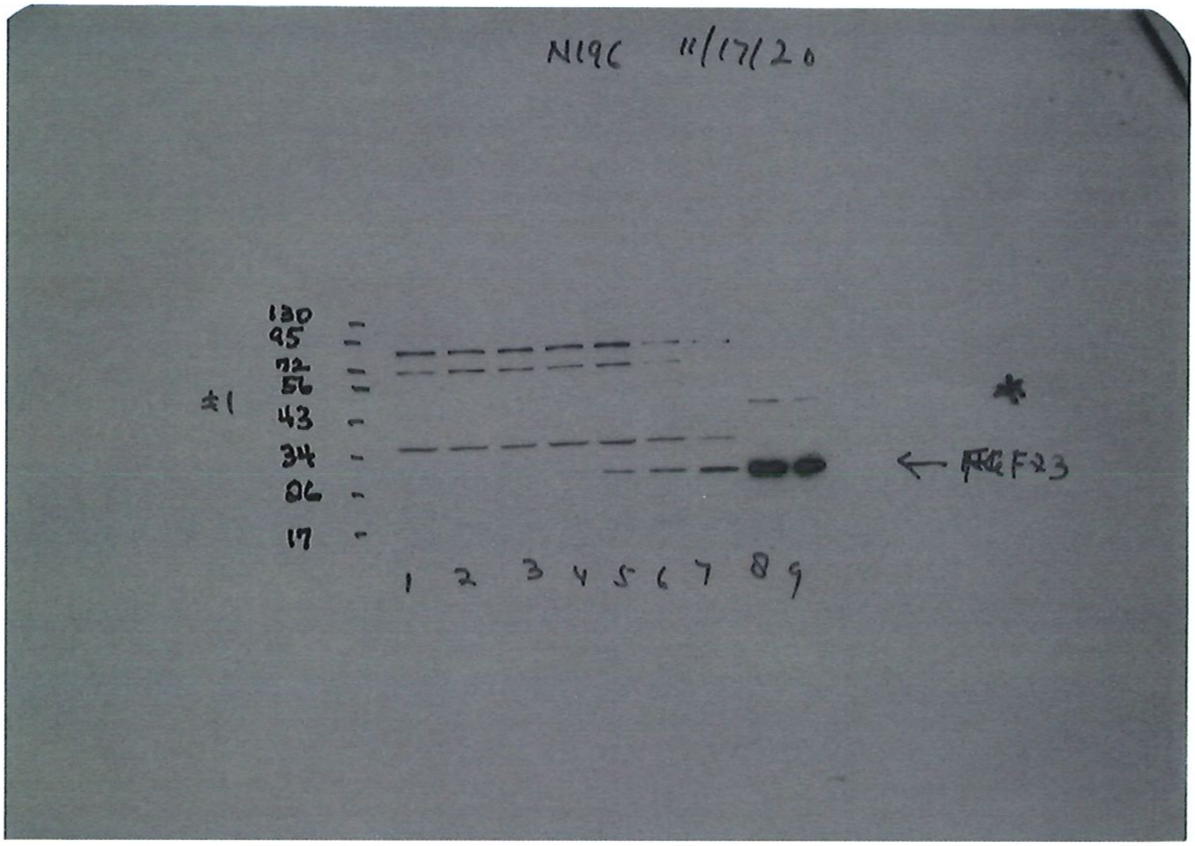

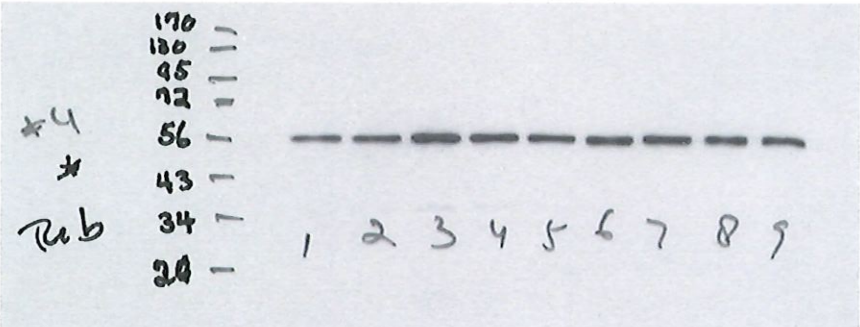

Supplement: S2 Fig — Unaltered radiographic images for Figs 1B and 2B. (PDF) [file pone.0264743.s002.pdf]
